# High-grade astrocytoma with piloid features: a case report of comprehensive management

**DOI:** 10.3389/fonc.2026.1819378

**Published:** 2026-05-29

**Authors:** Qun He, Fang Wang, Hua Cao, Zihuang Li, Mengqi Sun

**Affiliations:** 1 Department of Radiotherapy Shenzhen People’s Hospital, The Second Clinical Medical College, Jinan University, Shenzhen, China; 2 Medical Oncology, Shenzhen People’s Hospital, The Second Clinical Medical College, Jinan University, Shenzhen, China

**Keywords:** DNA methylation profiling, HGAP, high-grade astrocytoma with piloid features, radiotherapy, temozolomide

## Abstract

High-grade astrocytoma with piloid features (HGAP) is a rare and recently defined nervous system tumor. To date, clinical reports and therapeutic experience regarding HGAP remain scarce. Herein, we describe a 35-year-old female patient presenting with progressive numbness in the right neck and shoulder region for 2 months. Imaging examinations revealed an intramedullary tumor at the cervicomedullary junction. Histopathological analysis confirmed anaplastic glioma, and DNA methylation profiling established the diagnosis of HGAP. The patient subsequently underwent surgical resection combined with adjuvant radiotherapy and concomitant temozolomide chemotherapy. This tumor commonly occurs in the posterior cranial fossa of adults. In this report, we aim to present the clinical course of an extremely rare case of high-grade astrocytoma with piloid features (HGAP) that developed radiological progression in the early postoperative period, and to explore the clinical significance of this atypical manifestation.

## Introduction

High-grade Astrocytoma with Piloid features (HGAP) is a rare and aggressive brain tumor initially defined as “methylation class-anaplastic astrocytoma with piloid features (MC AAP)”. It was granted an independent classification in the 2021 World Health Organization (WHO) Classification of Tumors of the Central Nervous System due to its distinct tumor DNA methylation profiling characteristics (1, 2).This rare tumor most frequently arises in the posterior cranial fossa (PCF), particularly the cerebellum and brainstem regions, followed by the spinal cord and supratentorial compartment (3).This report aims to present the clinical course of an extremely rare case of high-grade astrocytoma with piloid features (HGAP) that developed radiological progression in the early postoperative period, and to explore the clinical significance of this atypical manifestation.

## Illustrative case

This case report was prepared in accordance with the CARE guidelines. A 35-year-old female patient was admitted due to progressive right neck and shoulder numbness for more than 2 months. Contrast-enhanced cervical magnetic resonance (MR) imaging revealed an intramedullary mass measuring 15mm × 14mm at the medullary-cervical junction, with ill-defined borders and heterogeneous signal intensity. The lesion presented as moderately hyperintense on T1-weighted imaging (T1WI) and heterogeneously hyperintense on T2-weighted imaging (T2WI), with marked heterogeneous enhancement after contrast administration and a few worm-like non-enhancing areas inside, which was highly suggestive of a neoplasm (**Figure 1**)

**Figure 1:**
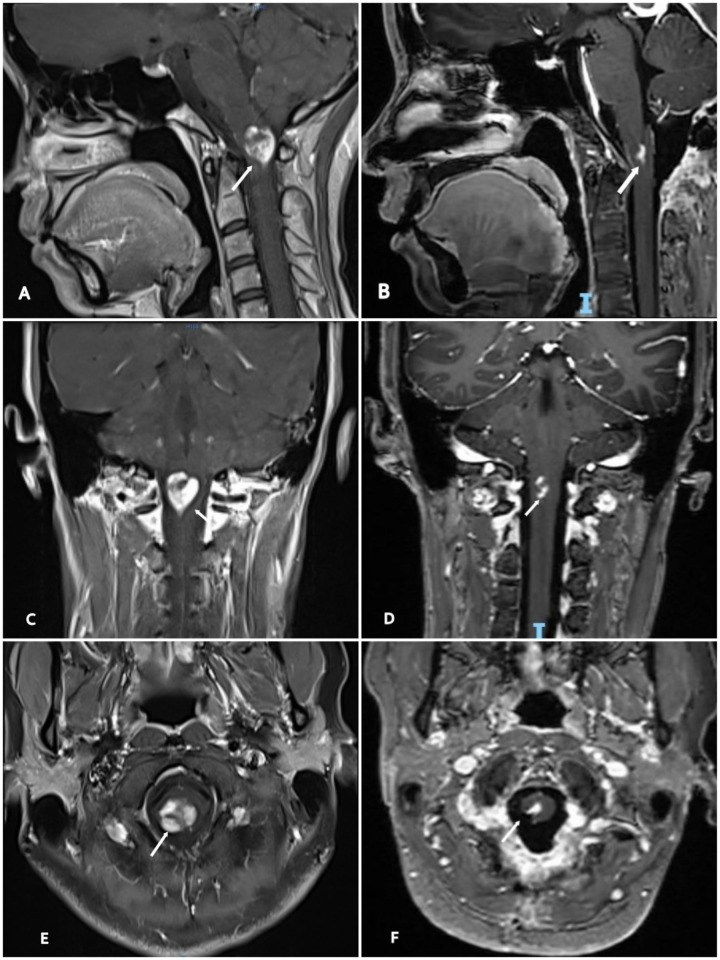
Preoperative and preradiotherapy MRI of the space-occupying lesion at the junction of the medulla oblongata and cervical spinal cord. Preoperative MRI: Sagittal **(A)**, coronal **(C)**, and axial **(E)** views demonstrate an in homogeneously enhancing lesion at the junction of the medulla oblongata and cervical spinal cord. Preradiotherapy MRI: Sagittal **(B)**, coronal **(D)**, and axial **(F)** views show near-total resection of the lesion with residual enhancement.

Postoperative pathological examination was performed on the medullary specimen. The clinical diagnosis was intracranial space-occupying lesion. Histopathological evaluation showed moderate tumor cellularity, significant nuclear atypia, rare mitotic figures, focal eosinophilic granular bodies, and microvascular proliferation is observed in the tumor parenchyma.(**Figure 2**) The IHC results were as follows: loss of ATRX protein expression (negative), IDH1-R132H negativity, retained nuclear expression of H3K27me3,a Ki-67 labeling index of 2%, p53 positivity in 30% of tumor cells, and BRAF-V600E negativity. (**Figure 3**) DNA methylation profiling indicated anaplastic astrocytoma with piloid features. Molecular testing demonstrated unmethylated MGMT promoter, homozygous deletion of CDKN2A and CDKN2B, and KIAA1549-BRAF gene fusion. Homozygous deletion of the CDKN2A/B genes and related gene fusions were detected using targeted next-generation sequencing (NGS).Data were analyzed using the Heidelberg Brain Tumor Methylation Classifier (German Cancer Research Center, DKFZ, v12.5).The calibrated score for this case was 0.76.Based on DNA methylation profiling and next-generation sequencing results (homozygous CDKN2A/B deletion and KIAA1549-BRAF fusion), the final integrated diagnosis was high-grade astrocytoma with piloid features (HGAP) (**Table 1**)

**Figure 2:**
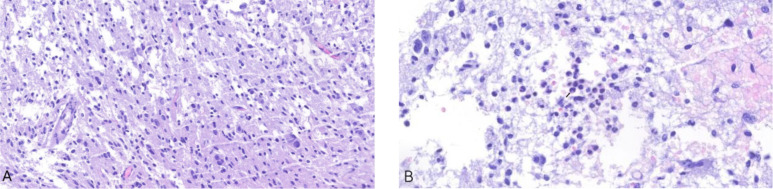
Histopathological features of the tumor. Hematoxylin and Eosin (H&E) staining of the lesion resected in December 2025 demonstrates moderate tumor cellularity, marked nuclear atypia, rare mitotic figures, focal formation of a few eosinophilic granular bodies, and microvascular proliferation is observed in the tumor parenchyma. **(A)** Original magnification ×200, medium power. **(B)** Original magnification ×400, high power.

**Figure 3:**
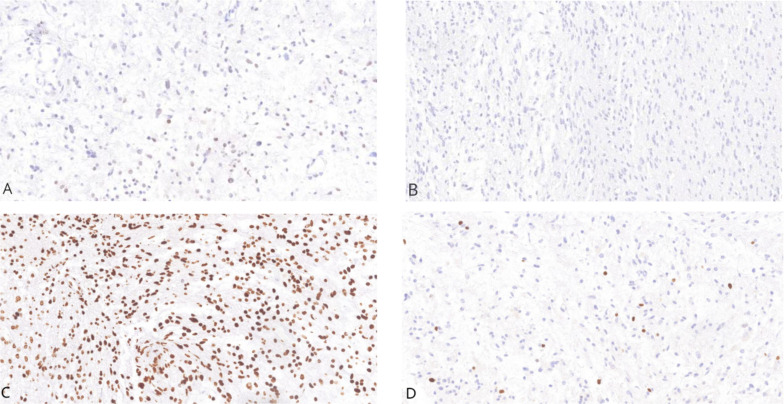
Immunohistochemical staining results. **(A)** Loss of ATRX expression (–); **(B)** IDH1-R132H negativity (–); **(C)**Retained nuclear expression of H3K27me3(+); **(D)** Ki-67 labeling index of 2%. Original magnification ×200, medium power for all panels.

**Table 1:** Analytical results of genome-wide DNA methylation profiling.

Test item	Result	Test item	Result
		PTEN gene mutation	No mutation
IDH1 gene R132 mutation	No mutation	PIK3CA gene mutation	No mutation
IDH2 gene R172 mutation	No mutation	SMARCB1 gene mutation	No mutation
Chromosome 1p/19q co - deletion	No deletion	SMARCA4 gene mutation	No mutation
TERT promoter C228T mutation	No mutation	PDGFRA K385 mutation	No mutation
TERT promoter C250T mutation	No mutation	PRKCA D463H mutation	No mutation
EGFR amplification	No amplification	FGFR1 gene mutation	No mutation
Chromosome 7 gain (+7)	No amplification	NF1 gene mutation	No mutation
Chromosome 10 loss (-10)	No deletion	NF2 gene mutation	No mutation
CDKN2A homozygous deletion	Deletion detected	CTNNB1 gene mutation	No mutation
CDKN2B homozygous deletion	Deletion detected	TSC1 gene mutation	No mutation
BRAF V600E mutation	No mutation	TSC2 gene mutation	No mutation
H3 - 3A (H3.3 K27M) mutation	No mutation	DICER1 gene mutation	No mutation
H3C2 (H3.1 K27M) mutation	No mutation	MET amplification	No amplification
H3C3 (H3.1 K27M) mutation	No mutation	MYCN amplification	No amplification
H3C14 (H3.2 K27M) mutation	No mutation	KIAA1549::BRAF fusion	Fusion detected
H3 - 3A (H3.3 G34) mutation	No mutation	ZFTA::RELA fusion	Not detected
ATRX gene mutation	No mutation	YAP1::MAMLD1 fusion	Not detected
TP53 gene mutation	No mutation	MN1::BEND2 fusion	Not detected
FUBP1 gene mutation	No mutation	NAB2::STAT6 fusion	Not detected
CIC gene mutation	No mutation	PTPRZ1::MET fusion	Not detected

One month after surgery, the patient underwent radiotherapy simulation. Pre-radiotherapy MRI showed: A small soft-tissue defect in the right posterior medulla filled with cerebrospinal fluid signal, consistent with postoperative changes; Marked enhancement at the surgical margin with a small focus of restricted diffusion (approximately 3mm × 3mm) (**Figure 1**). On physical examination, the patient was conscious with a Karnofsky Performance Status (KPS) score of 90, and no abnormalities were found on neurological and systemic examinations.

Radiotherapy was delivered using three-dimensional intensity-modulated radiotherapy (3D-IMRT). The gross tumor volume (GTV) was defined as the postoperative tumor cavity in the medulla on contrast-enhanced T1WI. The clinical target volume (CTV) was generated by a 1.0cm three-dimensional expansion of the GTV and constrained to the outer margin of the spinal cord. The planning target volume (PTV) was created by a 3mm expansion of the CTV. A total dose of 54 Gy in 27 fractions of 2 Gy was prescribed to cover 95% of the PTV. Concurrent chemotherapy with temozolomide 75 mg/m² per day was administered during radiotherapy, combined with dehydrative and symptomatic supportive care. The patient completed the entire course of radiotherapy without significant radiotherapy- or chemotherapy-related adverse events. Follow-up MRI performed 2 months after combined therapy showed a slight reduction in the marginal enhancement and the small restricted diffusion focus at the surgical cavity, with no other obvious abnormalities (**Figure 4**).

**Figure 4:**
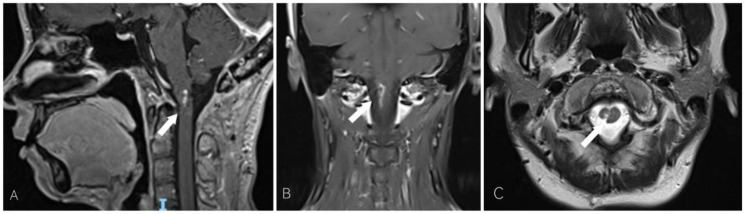
MRI of the space-occupying lesion at the medullary-cervical junction obtained 2 months after comprehensive treatment **(A)** Sagittal, **(B)** coronal, and **(C)** axial images show that the marginal enhancement at the surgical cavity and the small focal area of restricted diffusion are slightly reduced in extent compared with prior images.

## Discussion

High-grade astrocytoma with piloid features (HGAP) is a rare and aggressive tumor subtype established in the 2021 WHO Classification of Tumors of the Central Nervous System, first identified and named by the Reinhardt group in 2018 using DNA methylation profiling. Among 102 cases of anaplastic pilocytic astrocytoma, 83 cases presented a unique methylation signature, initially termed MC AAP and later uniformly renamed HGAP. HGAP has a low incidence, accounting for only 1%–3% of all brain tumors (4, 5), and mainly occurs in the posterior fossa. The age at onset ranges from 4 to 88 years, predominantly affecting adults and rarely children. Clinical manifestations are closely related to tumor location, including headache, dizziness, gait instability, limb weakness, sensory disturbance, visual impairment and urinary incontinence. Tumors located in the posterior fossa are prone to cause obstructive hydrocephalus and elevated intracranial pressure (6). Our patient presented with progressive right neck and shoulder numbness for over 2 months accompanied by headache and dizziness, which are typical manifestations of high cervical sensory impairment and consistent with neurological deficits caused by local mass effect.

Histopathologically, HGAP shows dual morphological features of pilocytic astrocytoma (PA) and glioblastoma with high heterogeneity, presenting both low-grade and high-grade characteristics (2, 7). Although no definite WHO grade is assigned, HGAP is clinically regarded as WHO grade 3–4 due to its invasiveness similar to IDH-mutant glioblastoma (2, 8, 9). Common histological features include increased cellularity, elevated mitotic activity, microvascular proliferation and pseudopalisading necrosis. Key molecular alterations include mitogen-activated protein kinase (MAPK) pathway abnormalities (NF1, FGFR, etc.), CDKN2A/B deletion, and ATRX mutation or loss (1). These features are diagnostically valuable but not specific to HGAP (2). In our case, the medullary lesion showed moderate cellularity, significant nuclear atypia, rare mitoses, and focal eosinophilic granular bodies, displaying a typical pathological spectrum of HGAP with mixed low-grade morphology and high-grade atypia, further supporting its morphological heterogeneity.

DNA methylation profiling is the gold standard for HGAP diagnosis, especially for cases with atypical morphology and rare locations. Methylation analysis indicated anaplastic astrocytoma with piloid features, and next-generation sequencing revealed homozygous CDKN2A/B deletion, KIAA1549-BRAF fusion, confirming the final diagnosis of HGAP (10). CDKN2A/B deletion is a core molecular marker for malignant invasion of HGAP (3, 11). KIAA1549-BRAF fusions have been described in approximately 19% of HGAP case. There are very few case reports describing the phenomenon of a PA transforming into an HGAP (12, 13). Although MGMT unmethylation indicates reduced temozolomide sensitivity, treatment can still follow the principles for high-grade gliomas (14). For cerebellar or NF1-related high-grade gliomas with ATRX mutation or CDKN2A/B deletion that do not meet other WHO criteria, methylation profiling is mandatory to exclude HGAP (3, 11). The Cimino group defined three methylation subtypes, among which gNF1 has the worst prognosis related to RNA processing dysregulation. 5Subtype stratification was unavailable in our case.

Imaging features of HGAP are heterogeneous without a unified standard. Typical findings include hypointensity on T1WI, hyperintensity on T2WI/FLAIR, heterogeneous enhancement with irregular rim enhancement, no obvious restricted diffusion on DWI, and high radiotracer uptake on PET-CT, which helps distinguish HGAP from glioblastoma. The Qudah group first reported finger-like/spiculated peritumoral enhancement and increased capillary permeability on DCE-MRI in 71% of intracranial HGAP cases (15). The Soni group reported rare leptomeningeal/dural dissemination, indicating tumor invasiveness (16). In our patient, cervical contrast MRI showed an intramedullary mass at the medullary-cervical junction with ill-defined borders, heterogeneous signals, moderate T1WI hyperintensity, heterogeneous T2WI hyperintensity and marked heterogeneous enhancement with worm-like non-enhancing areas. These features are consistent with solid-dominant heterogeneous enhancement of HGAP but differ from typical T1 hypointensity and spiculated peritumoral enhancement, suggesting site-specific imaging heterogeneity. Pre-radiotherapy MRI showed marginal enhancement and a 3 mm×3 mm restricted diffusion focus, considered early postoperative recurrence, which is rare in HGAP and provides a basis for precise radiotherapy target delineation.

No standardized treatment exists for HGAP, and maximal safe resection combined with adjuvant chemoradiotherapy is the main strategy. The tumor was located in the vital medullary-cervical junction, and surgery prioritized neurological function preservation. Radiotherapy with 3D-IMRT and concurrent temozolomide was administered one month after surgery, combined with dehydrative and supportive care. No obvious adverse events occurred, confirming the safety and feasibility of this regimen. Follow-up MRI at 2 months showed reduced enhancement and restricted diffusion without new lesions, achieving early radiological response and favorable short-term efficacy. Further follow-up will be performed at 4 months to evaluate long-term efficacy.

The Rubino group recommended exoscope-assisted resection for tumors in difficult regions such as the pineal gland (17). HGAP with ATRX mutation may be more sensitive to temozolomide (18), and radiotherapy is effective for cerebellar high-grade gliomas with low EGFR amplification (19, 20). FGFR, MEK and TRK inhibitors are potential targeted therapies for HGAP (20, 21), but their efficacy needs further validation. Only one case showed response to carboplatin plus vincristine (22). Advances in methylation profiling and sequencing support personalized therapy for HGAP.

The prognosis of HGAP is intermediate between benign PA and malignant IDH-wildtype GBM, with overall survival ranging from months to over 25 years and a 5-year survival rate of approximately 50% (2, 5). Due to the rarity of HGAP and limited single-center data, its molecular classification, prognostic markers, optimal targeted populations and radiotherapy standards for intramedullary lesions remain unclear. Large-scale multi-center studies are urgently needed to explore genotype-treatment correlations, establish diagnostic and therapeutic standards, and improve the prognosis of HGAP patients.

## Data Availability

The original contributions presented in the study are included in the article/supplementary material. Further inquiries can be directed to the corresponding authors.
